# Common variable immunodeficiency in two kindreds with heterogeneous phenotypes caused by novel heterozygous *NFKB1* mutations

**DOI:** 10.3389/fimmu.2022.973543

**Published:** 2022-09-20

**Authors:** Frederik Staels, Kerstin De Keukeleere, Matias Kinnunen, Salla Keskitalo, Flaminia Lorenzetti, Michiel Vanmeert, Teresa Prezzemolo, Emanuela Pasciuto, Eveline Lescrinier, Xavier Bossuyt, Margaux Gerbaux, Mathijs Willemsen, Julika Neumann, Sien Van Loo, Anniek Corveleyn, Karen Willekens, Ingeborg Stalmans, Isabelle Meyts, Adrian Liston, Stephanie Humblet-Baron, Mikko Seppänen, Markku Varjosalo, Rik Schrijvers

**Affiliations:** ^1^ Department of Microbiology, Immunology and Transplantation, Laboratory of Adaptive Immunology, KU Leuven, Leuven, Belgium; ^2^ Department of Microbiology, Immunology and Transplantation, Allergy and Clinical Immunology Research Group, KU Leuven, Leuven, Belgium; ^3^ Molecular Systems Biology Research Group and Proteomics Unit, Institute of Biotechnology, HiLIFE Helsinki Institute of Life Science, University of Helsinki, Helsinki, Finland; ^4^ Department of Pharmacy and Pharmacology, Medicinal Chemistry, Rega Institute for Medical Research, KU Leuven, Leuven, Belgium; ^5^ Department of Neurosciences, Research Group of Molecular Neurobiology, VIB-KU Leuven, Leuven, Belgium; ^6^ Department of Microbiology, Immunology and Transplantation, Laboratory of Clinical and Diagnostic Immunology, KU Leuven, Leuven, Belgium; ^7^ Center for Human Genetics, University Hospitals Leuven, Leuven, Belgium; ^8^ Department of Neurosciences, Research Group of Ophthalmology, KU Leuven, Leuven, Belgium; ^9^ Department of Microbiology, Immunology and Transplantation, Laboratory for Inborn Errors of Immunity, KU Leuven, Leuven, Belgium; ^10^ Department of Pediatrics, University Hospitals Leuven, Leuven, Belgium; ^11^ Laboratory of Lymphocyte Signaling and Development, Babraham Institute, Cambridge, United Kingdom; ^12^ Rare Disease and Pediatric Research Centers, New Children’s Hospital, University of Helsinki and Helsinki University Hospital, Helsinki, Finland; ^13^ Department of General Internal Medicine, University Hospitals Leuven, Leuven, Belgium

**Keywords:** primary immunodeficiency, functional validation, novel mutation, Nfkb1 (p50), haploinsufficiency

## Abstract

NFKB1 haploinsufficiengcy was first described in 2015 in three families with common variable immunodeficiency (CVID), presenting heterogeneously with symptoms of increased infectious susceptibility, skin lesions, malignant lymphoproliferation and autoimmunity. The described mutations all led to a rapid degradation of the mutant protein, resulting in a p50 haploinsufficient state. Since then, more than 50 other mutations have been reported, located throughout different domains of NFKB1 with the majority situated in the N-terminal Rel homology domain (RHD). The clinical spectrum has also expanded with possible disease manifestations in almost any organ system. In silico prediction tools are often used to estimate the pathogenicity of NFKB1 variants but to prove causality between disease and genetic findings, further downstream functional validation is required. In this report, we studied 2 families with CVID and two novel variants in *NFKB1* (c.1638-2A>G and c.787G>C). Both mutations affected mRNA and/or protein expression of NFKB1 and resulted in excessive NLRP3 inflammasome activation in patient macrophages and upregulated interferon stimulated gene expression. Protein-protein interaction analysis demonstrated a loss of interaction with NFKB1 interaction partners for the p.V263L mutation. In conclusion, we proved pathogenicity of two novel variants in *NFKB1* in two families with CVID characterized by variable and incomplete penetrance.

## Introduction

The nuclear factor of kappa light polypeptide gene enhancer in B cells (NF-κB) is crucial to several biological processes, including adaptive immune response, proliferation, cell survival and inflammation (1). The central players in this pathway consist of five transcription factors: REL-B, c-REL, REL-A/p65, NFκB1/p50 and NFκB2/p52. The latter two are produced as precursors which are processed by the proteasome to mature transcription factors p50 (from p105) and p52 (from p100) (1, 2). NF-κB signaling is activated *via* two pathways: the canonical and non-canonical (3). The canonical pathway is activated through different pattern recognition (PRR) and cytokine receptors leading to the activation of the IKKα/IKKβ/IKKγ complex and downstream degradation of IκBα, releasing transcription factors p50 and p65 [reviewed in (3)]. The non-canonical pathway is activated by lymphotoxin, receptor activator of NF-κB ligand (RANKL), CD40 ligand and B cell activating factor of the TNF family (BAFF) and mainly plays a role in induction of genes that orchestrate secondary lymphoid organ development [reviewed in (3)]. Not surprisingly, defects in NF-κB can lead to a spectrum of phenotypes ranging from variable immunodeficiency to autoinflammation (4). NFKB1 haploinsufficiency was first described in 2015 in three families with common variable immunodeficiency (CVID), presenting heterogeneously with symptoms of increased infectious susceptibility, skin lesions, malignant lymphoproliferation and autoimmunity (5). The described mutations all led to a rapid degradation of the mutant protein, resulting in a p50 haploinsufficient state. Since then, more than 50 other mutations have been reported, located throughout different domains of NFKB1, with the majority situated in the N-terminal Rel homology domain (RHD) (6). The clinical spectrum has also expanded with possible disease manifestations in almost any organ system (6). *In silico* prediction tools are often used to estimate the pathogenicity of *NFKB1* variants but to prove pathogenicity of a given variant, further downstream functional validation is required. In this report, we used immunophenotyping, functional analysis of peripheral blood mononuclear cells (PBMCs), RNA sequencing and mass spectrometry to validate two novel mutations in *NFKB1*, resulting in a phenotype of CVID with heterogenous inter- and intrafamilial presentation.

## Material and methods

### DNA extraction and genetic analysis

The Chemagen 4 K extraction kit (CMG-1074) was used according to the manufacturers’ instructions. Genomic DNA (gDNA)from the patients was isolated from whole blood. Targeted sequencing (Illumina Hiseq2500) for primary immunodeficiency (PID) genes was performed using a standard PID panel on Nimblegen v4 data. Annotation was done using RefSeq (release 78) and Alissa Interpret (v5.0). Pathogenic variants in *NFKB1* were verified by Sanger sequencing in index patients and family members.

### Computational modeling

The NFKB1 PDB structure was extracted from the RCSB database (7). The V263L mutation was introduced in the crystal structure using the built-in Pymol mutagenesis tool. To highlight the effect of the V263L mutation upon protein folding, the apo structure of both wild type (WT) and mutant NFKB1 were further processed for unrestrained molecular dynamics (MD) simulations. All preparative and unrestrained MD simulations in explicit solvent were run using the GPU version of the PMEMD engine provided with AMBER18 package. The ff14SB force field is used to describe the protein in the generated complex (8). Missing hydrogens and counter ions for neutralization are added through the LEAP module. Prior to the set-up, all systems are immersed into an octahedral box with TIP3P water molecules, spacing the atoms of the apo protein or protein complex 12 Å from the boundary of the simulation box edges. SHAKE algorithm is applied to all systems to constrain all bond lengths involving hydrogen atoms allowing a 2-fs time-step. The cut-off distance for van der Waals interaction is set at 12 Å. A system of Particle Mesh Ewald (PME) method is used to treat long-range electrostatic interactions. To remove steric clashes, a seven-step minimization procedure involving 1000 steps of steepest descent energy minimization is first performed followed by 4000 steps of conjugate gradient minimization at each step. The system is then slowly heated to 300 K after which the complexes are equilibrated by 2 ns position restraint MD simulations with 10.0 kcal/mol/Å^2^ constant force on the heavy atoms of protein and substrate under NPT condition (1 atm). After this equilibration protocol, unrestrained molecular dynamics is performed on all solvated systems under control of a Berendsen thermostat (1 atm) and a Langevin thermostat (300 K) using periodic boundary conditions applied in all three cartesian directions to mimic the infinity of the system. The nonbonded list is updated every 25 steps, with periodic new random number seeds to prevent simulation synchronization of the trajectories. The trajectories are sampled every 2 ps for analysis in production dynamics. Unrestrained 100 ns MD simulations were finally carried out under NPT conditions. To estimate the stability of the WT compared to the mutant NFKB1 of the ligand with the protein target, the cpptraj module in AmberTools18 was used to additionally calculate root-mean-squared deviation (RMSD) on Cα atoms from the average structure from the MD simulation.

### PBMC isolation

Whole blood was diluted 1:1 with RPMI 1640 and layered over lymphocyte separation medium (LSM) (MP Biomedicals, 0850494-CF). Tubes were centrifuged at 400 x g for 25 minutes and PBMCs were harvested and stored in liquid nitrogen until FACS staining or used immediately for functional assays.

### Western blot

Fresh or thawed PBMCs were plated overnight, and afterwards stimulated for indicated time points with 50 ng/ml+ 1 µg/ml PMA/ionomycin (Sigma-Aldrich) in RPMI 1640 medium (supplemented with FBS 5% + HEPES+ MEM non-essential aminoacids) (GibcoTM). After stimulation, PBMCs were lysed in lysis buffer [50 mM Tris-HCl pH 7.5, 135 mM NaCl, 1.5 mM MgCl2, 1% Triton-X, 10% glycerol, 1X protease inhibitor (Pierce TM Protease Inhibitor, ThermoFisher Scientific) and 1X phosphatase inhibitor (PhosSTOP, Roche)]. Protein concentrations were determined using a Bradford Protein Assay (Bio-Rad). Protein lysate was denaturized in LDS (NuPAGE LDS sample buffer, Novex) and DTT (Bio-Rad) at 70°C for 10 min and was then loaded on a 4-12% Bis-Tris polyacrylamide gel (BoltTM Bis-Tris Plus, Thermo Fisher Scientific) in MOPS buffer (NuPAGE MOPS SDS running buffer, Novex). Separated proteins on the gel were transferred onto a methanol-activated PVDF membrane (GE Healthcare) in transfer buffer [10% methanol, 1X Tris/Glycine Buffer (Bio-Rad)]. After transferring, the PVDF membrane was blocked in 5% milk TBS-T for 30 min at RT and then incubated with primary antibody O/N at 4°C followed by a wash with TBS-T and incubation with a secondary antibody for 1 h at RT. Primary antibodies used for Western blot: rabbit anti-p105/p50 (Cell Signaling Technology, D4P4D), rabbit anti-p-p105 (Cell Signaling Technology, 18E6) and mouse anti-GAPDH (Invitrogen). Secondary antibodies used were conjugated with HRP: goat anti-rabbit (Abcam) and anti-mouse (Rockland). Secondary antibodies were visualized using ECLTM15 Prime Western Blotting Detection Reagent (AmershamTM) with the G:box Chemi-XRQ and quantified using ImageJ.

### mRNA isolation and generation of cDNA

mRNA was isolated from PBMCs using Trizol reagent (Life Technologies). 500 µL of trizol was added to PBMCs or differentiated macrophages and frozen at -80°C. Later, samples were thawed for further processing. 100 µL chloroform was added and cells were centrifuged (10000 x g) for 15 min at 4°C. Aqueous solution was collected, 1 µL Glycoblues and 200 µL isopropanol were added and samples were incubated at RT for 15 min. Thereafter, samples were centrifuged (10000 x g) for 15 min at 4°C, supernatant was removed, 500 µL of 75% ethanol was added, samples were again centrifuged (10000 x g) for 15 min at 4°C, supernatant was removed and samples were air-dried for 10 min. Finally, the pellet was resuspended in 15 µL elution buffer (MACHEREY-NAGEL). Complementary DNA (cDNA) was synthesized from RNA using the GoScriptTM Reverse Transcription System (Promega) *via* RT-PCR (SimpliAmp Thermal Cycler, ThermoFisher Scientific).

### (q)PCR

Forward and reverse primers were purchased from Integrated DNA Technologies (IDT). For *NFKB1* forward primer AACAAGAAGTCTTACCCTCAGGT and reverse primer AGATCCCATCCTCACAGTGTTTT were used. For IL1B we used forward primer TGGCAATGAGGATGACTTGT and reverse primer GGAAAGAAGGTGCTCAGGTC. *HPRT1* was used as housekeeping gene for all performed qPCR’s. The reaction was performed as followed: 1 µl of primers (0.75 µM), 8 ng of DNA (2 ng/µl) and 1X Fast SYBR TM Green Master Mix (ThermoFisher Scientific) were mixed and plated on a 96-well plate. The plate was run on the StepOneTM Real-Time PCR system (ThermoFisher Scientific) and analyzed with StepOne Software v2.3. For detection of any alternative spliced product in the context of the c.1638-2A>G mutation, we performed a PCR reaction using a forward primer TGGTGAGGTCACTCTAACGTA and reverse primer GGGGTGTGGTTCCATCGTAG. 10 µM of primers and 10 ng of cDNA were added to a PCR reaction mix containing 1X Q5 reaction buffer (New England Biolabs), 10 mM dNTP (Promega), Q5 high GC enhancer (New England Biolabs) and Q5 hot start high fidelity polymerase (New England Biolabs). PCR cycling was performed at the following parameters: One cycle of denaturation (98°C for 30 s), 35 three-segment cycles of amplification (98°C for 10 s, 62°C for 30 s and 72°C for 30 s) and a final cycle of extension (72°C for 2 min). 1X DNA loading dye (Thermo Scientific) was added to the PCR products and loaded on a 1% agarose gel (VWR Life Science). PCR bands were visualized with the Gbox ChemiXRQ.

### Macrophage differentiation and NLRP3 inflammasome activation

PBMCs, isolated as described in the section PBMC isolation, were used for monocyte purification using negative selection for CD14++CD16- monocytes (Miltenyi, 130-117-337) according to the manufacturer’s protocol. Before plating, purity of the samples was evaluated on flowcytometry by CD14 staining (>80% of total cells). Monocytes were counted, resuspended in macrophage serum free medium (Gibco, 12065-074) and seeded in a 96 well plate at a density of 1x10^6^ cells/ml in 100 µL with GM-CSF 10 ng/ml (Biolegend, 572902). Medium was changed every 3 days and macrophages were harvested at day 7. Macrophages were primed with lipopolysaccharide (LPS, 1 µg/ml; Sigma) for 6 h to induce the expression of IL1B, and thereafter activation of the NLRP3 inflammasome was induced with adenosine triphosphate (ATP, 5 mM, 45 min). Secretion of the mature IL-1β was measured by ELISA (Abcam, ab46052) according to manufacturer´s recommendations. Cells were further used for RNA extraction as described in the section isolation of mRNA and generation of cDNA.

### Immunophenotyping of B, NK cells and monocytes

PBMCs were isolated from heparinized blood samples of human healthy donors using Ficoll-Paque density centrifugation (MP biomedicals), frozen and then stored in liquid nitrogen. Frozen PBMCs were thawed and counted, and cell concentration was adjusted to 5 x 10^6 for each sample.

Cells were plated in a V-bottom 96-well plate, washed once with PBS (Fisher Scientific) and stained with live/dead marker (fixable viability dye eFluor780, eBioscience) and fluorochrome-conjugated antibodies against surface markers (**Supplementary Table E1**) to identify the following subsets: T cells (CD3^+^, CD4^+^ and CD8^+^), B cells (total CD3^-^CD19^+^, naïve CD27^-^, memory CD27^+^, switched memory CD27^+^IgM^-^IgD^-^, transitional CD24^+^CD38^+^ and CD21^low^ B cells), NK cells (total CD3^-^CD56^+^, CD56^bright^CD16^-/+^, CD56^dim^CD16^+^ NK cells) and monocytes (classical CD14^+^CD16^-^, intermediate CD14^+^CD16^+^ and non classical CD14^-^CD16^+^ monocytes).

Samples were stained for 60 min at 4°C, washed twice in PBS/1% FBS (Tico Europe), and then fixed. Cells were stored overnight at 4°C and were then acquired on a Symphony flow cytometer with Diva software (BD Biosciences). A minimum of 5 x 10^5 events were acquired for each sample. Compensation beads (UltraComp eBeads, ThermoFisher) were used to optimize fluorescence compensation settings for multi-color flow cytometric analysis at a Symphony flow cytometer.

### IκBα degradation

Fresh whole blood (lithium heparin) was incubated with 10 µg/mL polymyxine B (Sigma-Aldrich), CD45-V500 (BD biosciences), and CD14-FITC (BD biosciences) in the absence or presence of 75 ng/mL IL-1b (eBioscience), 1 µg/ml Pam3CKS4 (*In vivo*gen), 3 µg/mL R848 (*In vivo*gen), or 20 ng/mL TNF-a (Peprotech). Cells were incubated for 15 min at 37°C, 5% CO2. Thereafter, the RBCs were lysed, fixed [with Lyse/Fix Buffer (BD biosciences) for 10 minutes at 37°C, 5% CO2], permeabilized (Perm Buffer II, BD biosciences) for 30 minutes on ice, washed with Stain buffer (BD biosciences) and incubated with 1:20 diluted PE Mouse anti-IkBa (BD biosciences).

### Plasma and lacrimal fluid cytokine measurement

Plasma cytokines were determined using a multiplex assay (MSD multi spot assay system, proinflammatory panel 1), according to the manufacturer’s instructions.

### NanoString analysis

Direct digital detection of mRNA levels of selected genes from patients’ PBMCs was performed using nCounter^®^ Analysis System (NanoString Technologies) as described in (9).

### Mass spectrometry (affinity purification and Biotin proximity ligation assay (BioID)

Interaction analysis was done following the workflow detailed by Liu et al. (10, 11). The NFKB1 constructs were expressed with N-terminal MAC-tags and three biological replicates were prepared for both AP-MS and BioID, for each cell line.

2μl of the reconstituted samples were analyzed using the Evosep One liquid chromatography system coupled to a hybrid trapped ion mobility quadrupole TOF mass spectrometer (Bruker timsTOF Pro) *via* a CaptiveSpray nano-electrospray ion source. An 8 cm × 150 µm column with 1.5 µm C18 beads (EV1109, Evosep) was used for peptide separation with the 60 samples per day method (21 min gradient time). Mobile phases A and B were 0.1% formic acid in water and 0.1% formic acid in acetonitrile, respectively. The MS analysis was performed in the positive-ion mode using data-dependent acquisition (DDA) in PASEF mode with 10 PASEF scans per topN acquisition cycle.

Raw data acquired in PASEF mode were processed with Fragpipe version 17.1, including MSFragger version 3.4, against the human entries of Uniprot database (UP000005640, 20371 entries). Carbamidomethylation of cysteine residues was used as static modification. Aminoterminal acetylation and oxidation of methionine were used as the dynamic modification. Trypsin was selected as enzyme, and maximum of two missed cleavages were allowed. Label-free quantification parameters were left to default settings. Peptides with false discovery rate (FDR) < 0.05 were exported from the search results.

The resulting protein detections were filtered using SAINT and MAC-tagged GFP control interactions. Interactors with < 0.01 BFDR were chosen as the filtered high-confidence interactors. The triplicate samples were individually bait normalized by the NFKB1 spectral counts and the normalized average spectral counts were then used to made dotplots with ProhitsViz.The filtered BioID results were used to run GO biological process term enrichment analysis using the DAVID bioinformatics resources (PMID: 19131956).

### Statistical analysis

Data with ≥2 independent experiments were pooled (if available) for statistical analysis using a Mann-Whitney U test.

## Results

### Identification of *NFKB1* variants in 2 families with variable phenotypes of immunodeficiency, lymphoproliferation and autoinflammation

With next generation sequencing, we studied a cohort of patients with broad immunological phenotypes in the adult immunology clinic. Targeted sequencing with a PID panel revealed the presence of 2 heterozygous *NFKB1* variants in 2 different families. The clinical and immunological characteristics are summarized in **Tables 1**, **2**.

**Table 1:** Demographic and clinical characteristics of all NFKB1 carriers.

Family	Family 1		Family 2				
NFKB1 carrier	*1.*II.1	*1.*III.1	*2.*II.1	*2.*II.2	*2.*II.3	*2.*III.1	*2.*III.2
Clinically affected	+	+	+	–	–	+	+
Age at genetic diagnosis	47 y	18 y	56 y	65y	60y	27y	20y
Age of onset	Childhood-adolescence	Adolescence	56 y	NA	NA	25y	32y
Infectious susceptibility	+ (URTI, LRTI, pyelonephritis)	+ (URTI)	–	–	–	+ (URTI, bilateral pneumonia)	–
Autoimmunity or inflammation	+ (psoriasis, psoriatic arthritis)	–	+ (polymyalgia rheumatica)	–	–	? Bilateral unexplained keratitis	–
Lymphoproliferation	+ (splenomegaly, diffuse abdominal and intrathoracic adenopathy)	–	–	–	–	–	+ (reactive hyperplasia, cervical adenopathy)
Gastrointestinal	+ (enteropathy with normal biopsy)	–	–	–	–	–	–
Pulmonary function test	Low diffusion capacity (TLCO 74-82% predictive value)	Normal	Normal	Normal	Normal	Normal	ND
Abdominal ultrasound	Splenomegaly	Normal	Normal	ND	ND	Hepatomegaly (16 cm midclavicular)	ND
Echocardiography	Normal	ND	ND	Normal	ND	ND	ND
Chest radiograph or CT scan	Intrathoracic adenopathy on CT scan	Normal CXR	Normal CT scan	Normal CT scan	Bronchial wall thickening right lower lobe on CT scan	Normal CT scan (prominent mediastinal adenopathy)	Normal CT scan
Endoscopy	Normal colonoscopy and gastroscopy	Normal colonoscopy and gastroscopy	ND	ND	ND	Normal gastroscopy	ND
Biopsy	Liver: nonspecific hepatitis Lymph node: increase in PD1 positive T cells and B cell immunoblasts in the interfollicular region Colon/stomach: normal	Stomach/colon: normal	ND	ND	ND	ND	Lymph node: reactive hyperplasia
Treatment	SCIG Sulfasalazine (stopped 2011 because of liver function test disorder) Methotrexate (stopped 2016 because of remission and liver function test disorder)	/	IVIG Prednisone (cessation after clinical remission)	/	/	SCIG	/

URTI, upper respiratory tract infection; LRTI, lower respiratory tract infection; SCIG, subcutaneous immunoglobulins; IVIG, intravenous immunoglobulins; ND, not determined; NA, not applicable. Bold values are outside of the reference range values.

**Table 2:** Laboratory analysis of all NFKB1 carriers at first evaluation in the outpatient immunology clinic.

Family		Family 1		Family 2				
NFKB1 carrier		*1*.II.1	*1*.III.1	*2*.II.1	*2.*II.2	*2*.II.3	*2.*III.1	*2*.III.2
**Hemoglobin**	12-16g/dl	**11.7-**13	**11.9**-14.4	14.4-15	**10.1-11.4**	15.2-16.5	**11.7**-12.9	15.2-16
**Thrombocytes**	150-400x10^9^/L	**77-124**	247-315	217-266	**140**-425	224-232	165-270	229-295
**Leukocytes**	4-11x10^9^/L	**2.43-3.84**	5.5-8.33	4.44-7.71	9-**23**	5-6.62	**3.5**-5	**3.93**-6.77
**B cells (CD19+)**	0.082-0.476 x10^9^/L	**0.038**	0.269	**0.079**	**0.055**	0.277	**3.2**	0.156
**smBcells (CD27+IgM-IgD-)**	% of CD19	**Not detectable**	8.6	**Not detectable**	**Not detectable**	3.2	**Not detectable**	**Not detectable**
**T cells (CD3+)**	0.798-2.823 x10^9^/L	0.799	2.213	1.090	1.079	1.942	1.364	1.174
**CD4+ T cells**	0.455-1.885 x10^9^/L	0.599	1.285	0.638	0.760	1.162	0.760	0.513
**CD8+ T cells**	0.219-1.123 x10^9^/L	0.255	1.281	0.413	0.314	0.719	0.533	0.623
**HLA-DR+ T cells**	≤18% of CD3	**64.1**	8.5	7.4	5.4	11.2	ND	ND
**Naïve T cells (CD45RA+CD27+)**	%	6.4	70.7	42.1	70.9	69.5	40.1	ND
**αβ-TCR (**	≥90% of CD3	98.84	93.72	96.16	98.81	94.76	ND	ND
**γδ-TCR**	≤10% of CD3	1.3	5.9	3.4	0.8	5.5	ND	ND
**NK cells (CD3-CD56+)**	0.066-0.745x10^9^/L	0.190	0.109	0.310	0.283	0.206	**0.033**	0.188
**IgG**	7.51-15.6 g/L	**0.45**	**6.26**	**2.89**	**6.52**	9.04	**0.65**	9.03
**IgM**	0.46-3.04 g/L	**0.06**	**0.38**	**0.17**	0.49	1.13	**0.05**	**0.37**
**IgA**	0.82-4.53 g/L	**<0.07**	1.16	**0.32**	1.25	2.35	**<0.07**	0.91
**Pneumococcal response^§^**	Normal/abnormal	**Abnormal**	**Abnormal**	**Abnormal**	ND	Normal	**Abnormal**	ND
**Mitogen T cell proliferation**	Normal	ND	Normal on PHA	Normal to candida, tetanus toxoid, herpes simplex and CMV	Normal to tetanus toxoid and PHA	Normal to tetanus toxoid and PHA	Normal to tetanus toxoid	ND
**Autoantibodies (ANF, ANCA)**	Positive/negative	Negative	ND	Negative	ND		ND	ND

^§^Interpretation based on AAAAI (fully) criteria and (24). Bold values are outside of the reference range values. ND, not determined.

**Figure 1:**
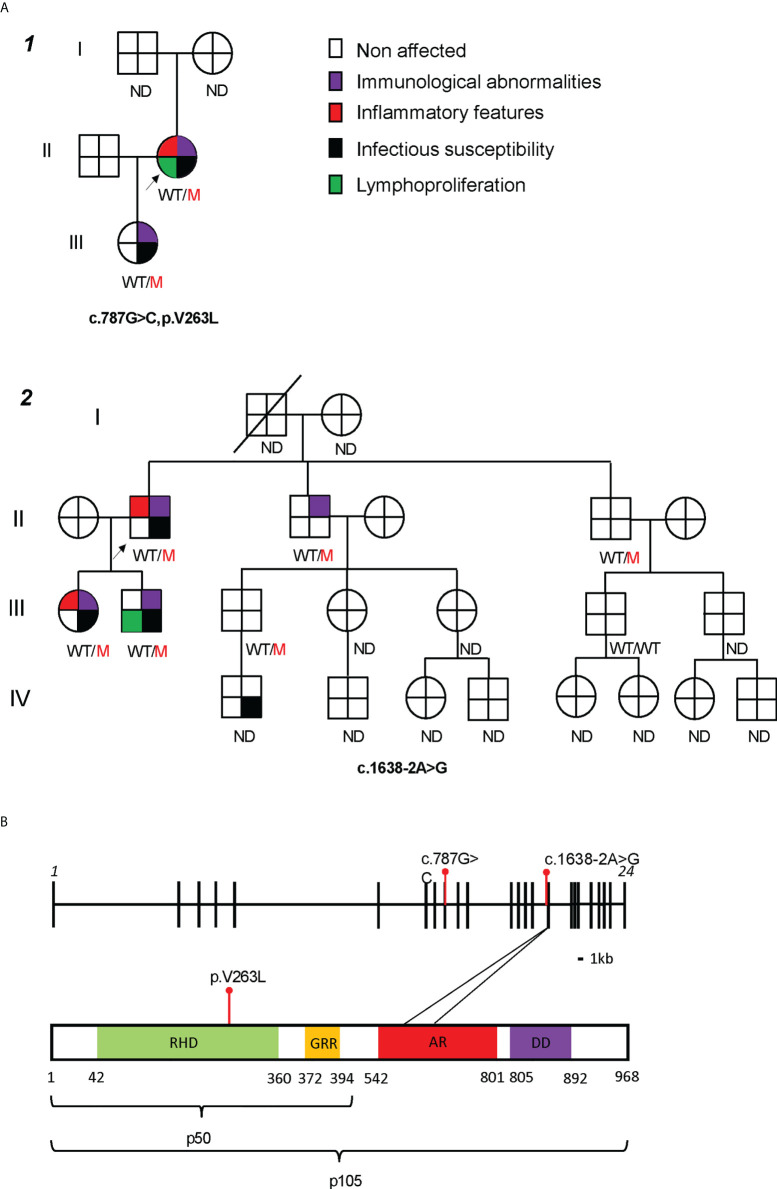
Characterization of two families with mutations in *NFKB1.* **(A)** Family pedigrees, black arrow pointing the index patients. ND not determined. **(B)** NFKB1 gene (up) and protein (down) structure, with studied mutations in red. RHD, Rel Homology Domain; GRR, glycine rich repeat; AR, ankyrin repeat; DD, death domain. *p < 0.05, **p < 0.01, ***p < 0.001, ****p < 0.0001 by Mann-Whitney U test.

Family 1 (**Figure 1A**, **Supplementary E1A**) included 2 affected members who presented with recurrent respiratory tract infections and B cell dysfunction. The 47-year-old index patient (*1*.II.1) met the ESID criteria for CVID and had a more severe course of disease characterized by hospitalizations for pneumonia and pyelonephritis. In addition, as part of the non-infectious CVID spectrum, she suffered from psoriatic arthritis (treated with sulfasalazine and afterwards methotrexate, both stopped before evaluation of CVID because of liver function test disorders), non-malignant intra-abdominal and thoracic lymphoproliferation at the age of 47 with coexistent chronic diarrhea at that time. Lymph node biopsy showed expansion of T and B cells in the interfollicular region but no signs of malignancy; gastro-intestinal biopsies (duodenal, colon) were unremarkable (**Table 1**). Eventually these symptoms spontaneously resolved without the need of immunomodulating agents. In immunophenotyping, she had low B cells, low switched memory B cells, hypogammaglobulinemia and abnormal pneumococcal vaccine response (**Table 1**). Her 19-year-old daughter (*1.*III.1) only displayed mild infections never requiring hospitalization, decreased IgG levels and abnormal pneumococcal vaccine response, with normal B cells. Genetic analysis by targeted, also Sanger-confirmed sequencing showed a heterozygous c.787G>C (p.V263L) mutation in *NFKB1*.

Family 2 (**Figure 1A** and **Supplementary E1A**) included 4 affected members with variable presentation (clinical or subclinical). The index patient (*2*.II.1) was diagnosed with CVID at the age of 55 after investigations for polymyalgia rheumatica. Laboratory results before start of corticosteroids showed marked hypogammaglobulinemia, low switched memory B cells and no responses to pneumococcal vaccine. He did not report any severe infections during child- or adulthood. His 30-year-old daughter (*2*.III.1) presented at the age of 26 with recurrent sinopulmonary infections requiring antibiotic treatment. She had hypogammaglobulinemia, low switched memory B cells, and no responses to pneumococcal vaccine on laboratory evaluation. At the age of 28, she developed a hitherto unexplained bilateral keratitis refractory to diverse lubricants and ultimately treated with punctal plugs. His 31-year-old son (*2*.III.2) had a history of recurrent cervical adenopathy at the age of 20 years, for which he underwent an excision biopsy showing reactive hyperplasia. Infectious and autoimmune serology at that time was negative. Laboratory analysis revealed low IgM but normal IgG and IgA. Both siblings from the index patient (*2.*II.2 and *2.*II.3) were asymptomatic and laboratory results only showed abnormalities in *2*.II.2 with isolated hypogammaglobulinemia. Targeted sequencing in the index showed a heterozygous splicing mutation c.1638-2A>G, located before exon 16. Sanger sequencing confirmed the presence of this mutation in both his children and siblings.

The two mutations identified in *NFKB1* affected different domains of the protein structure (**Figure 1B**). The c.787G>C mutation (Family 1) led to a substitution of a conserved valine to a leucin at the 263 residue (p.V263L) in the Rel Homology Domain (RHD) of the NFKB1 protein. An intronic mutation in Family 2, c.1638-2A>G, was located before exon 16. According to the database splicing consensus single nucleotide variant (dbscSNV) the intronic mutation was predicted to alter splicing (ADA score 0.99 and RF score 0.94). Both mutations were not previously reported in public genomic databases (gnomad, ExAC and 1000 genomes). Based on *in silico* prediction scores (CADD, PolyPhen 2, SIFT), they were predicted to be damaging to the protein structure or function (**Supplementary E1B**, CADD score). Structural modelling of the p.V263L mutant showed a disruption of the C-terminal β-barrel (**Supplementary E1C**, right panel, dotted circle 1) which could trigger an unfolding event. Additionally, an upstream loop is converted into a twisted β-sheet exposing hydrophobic residues to the water interface which could promote protein aggregation (**Supplementary E1C**, right panel, dotted circle 2). Furthermore, a single-turn α-helical structure is destabilized. In conclusion, these findings predict that both mutations are likely deleterious.

### Effect on transcriptional and protein expression of NFKB1 and analysis for upstream defects in canonical NF-κB signaling

**Figure 2:**
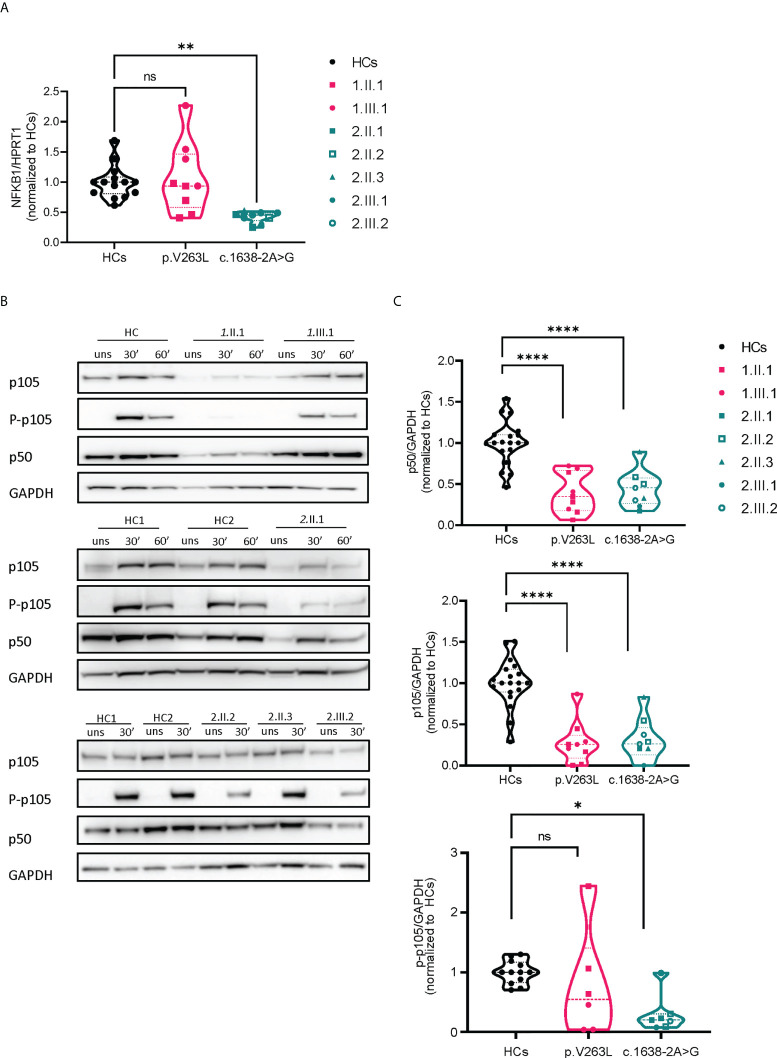
mRNA and protein expression analysis of both *NFKB1* mutations. **(A)** Relative mRNA expression levels by qPCR, corrected for housekeeping (HPRT1). Dots represent technical duplicates from separate experiments (each with a minimum of 2 healthy controls) **(B)** Representative Western blots, stimulation time points 30’ and 60’ with PMA/ionomycin (50 ng/ml + 1 µg/ml) **(C)** Quantification of image bands by ImageJ software, dots represent different experiments, normalized to the respective mean of healthy control(s). Statistics were applied if ≥2 independent experiments were performed (see methods).

**Figure 3:**
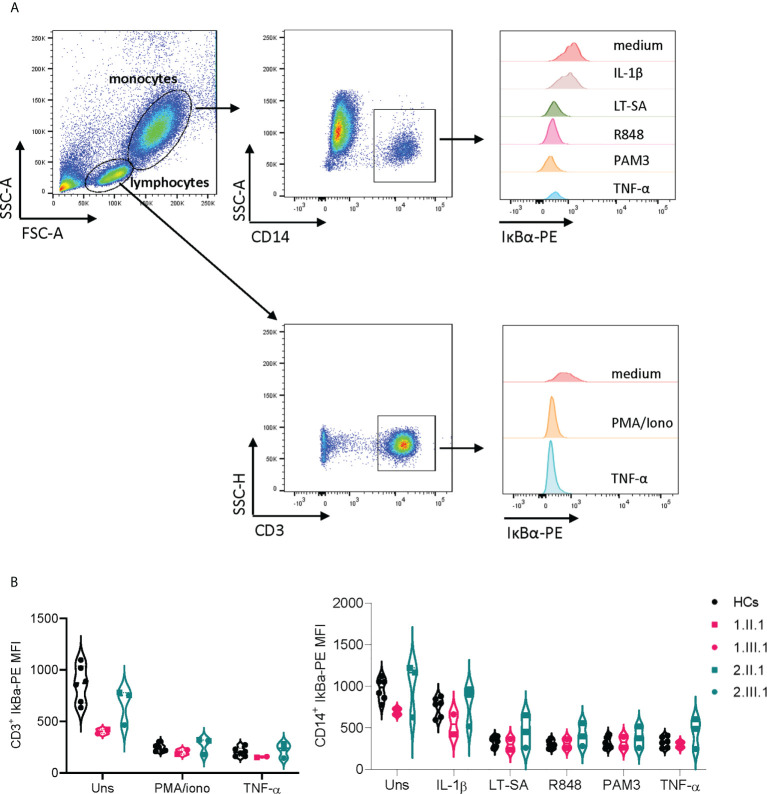
IκBα degradation in T cells and monocytes is unaffected in NFKB1 mutation carriers. **(A)** Gating strategy on CD3^+^ and CD14^+^ cells **(B)** Mean fluorescence index (MFI) of IκBα-PE in CD3^+^ T cells and CD14^+^ monocytes of NFKB1 patients and healthy controls. For patient 2.III.1, experiment was repeated twice.

We assessed the transcriptional levels of *NFKB1* by qPCR and protein expression of p50/p105 in PBMCs of affected and unaffected family members (**Figures 2A–C** and **Supplementary E2**). Phosphorylation of p105 was examined after stimulation of PBMCs with phorbol myristate acetate and ionomycin. In the first family, the p.V256L mutation had no overall effect on mRNA expression, although there was considerable interindividual variation. The index patient *1.*II.1, with the most severe phenotype, had slightly reduced levels (75% compared to healthy controls), while her daughter who was mildly affected produced normal levels of NFKB1 mRNA (**Figure 2A**). Protein levels of p50/p105 were reduced in both of them, albeit more significantly in *1*.II.1 compared to healthy controls. Phosphorylation of p105 was reduced only in *1*.II.1 (**Figures 2B, C**). In the second family, all carriers of the splicing variant c.1638-2A>G had 30-50% of transcriptional levels compared to healthy controls, suggesting nonsense mediated decay of a putative alternatively spliced mRNA (**Figure 2A**). An alternatively spliced product was not detected by PCR on cDNA using primers spanning exon 14-19 (**Supplementary E3**). As expected, protein levels of p50/p105 were reduced in all patients, except for *2.*II.3, who was unaffected (**Figures 2B, C**). Phosphorylation of p105 was reduced in all members (*2*.III.1 not tested), except for the unaffected member *2*.II.3. To study if our NFKB1 haploinsufficiency patients had any upstream defect in canonical NF-κB signaling, we assessed IκBα degradation on CD3+ T cells and CD14^+^ monocytes, using different stimuli, as a surrogate marker (**Figures 3A, B**). These experiments showed that IκBα was degraded to a similar degree in all patients compared to healthy controls.

### Increased NLRP3 inflammasome activity and interferon (IFN) signature in NFKB1 V263L and c.1638-2A>G affected individuals

**Figure 4:**
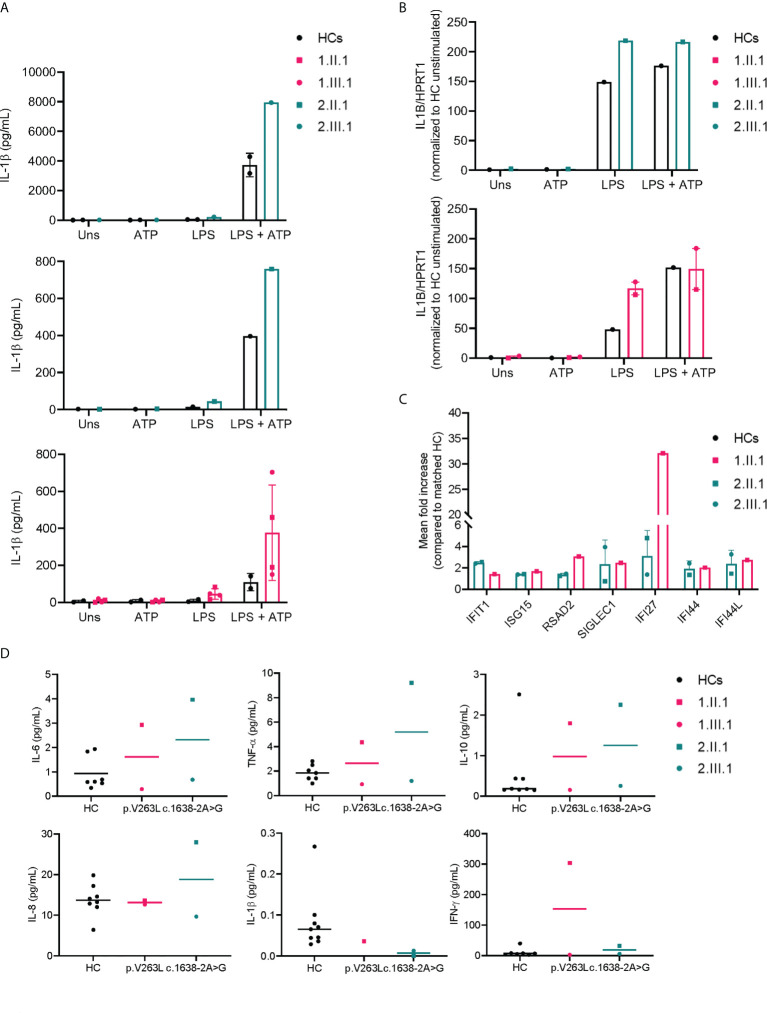
Increased NLRP3 inflammasome activity and IFN signature in NFKB1 affected individuals. **(A)** IL-1β secretion by macrophages of NFKB1 patients and healthy controls (=1-2 for each experiment) measured by ELISA, LPS 10 μg/mL (for *2.*III.1) or 1 μg/mL (all other subjects) was used for priming during 6 hours followed by ATP 5mM for 45 minutes **(B)** IL1B transcript levels from macrophages by qPCR **(C)** ISG expression relative to a matched healthy control (n=1 for each patient) **(D)** plasma cytokine measurements by multiplex analysis.

We assessed NLRP3 inflammasome activity on differentiated macrophages both by qPCR (*IL1B* transcriptional level) and ELISA (IL-1β secretion) of affected members in both families (**Figures 4A, B**), and interferon stimulated gene (ISG) expression by NanoString on PBMCs (**Figure 4C**). Serum cytokines were determined on plasma samples (**Figure 4D**). In family 1, only the index patient (*1.*II.1) had inflammatory symptoms (psoriatic arthritis), while her daughter did not present with any inflammatory feature (till present). NLRP3 inflammasome activity upon stimulation of differentiated macrophages was higher in both patients (*1.*II.1, *1*.III.1). ISG signature was only determined for the index patient (*1.*II.1) and upregulated compared to a matched healthy control. Consistent with an inflammatory phenotype, the majority of proinflammatory cytokines (IL-6, TNFα and IFN-γ) were increased in patient *1*.II.1, while remaining normal in patient *1*.III.1. Two affected patients of family 2 (*2.*II.1 and *2*.III.1) had inflammatory symptoms (polymyalgia rheumatica and bilateral idiopathic keratitis). NLRP3 inflammasome activity was persistently higher for both compared to healthy controls. Serum cytokine measurements showed comparable levels of proinflammatory cytokines IL1β, IL-6, TNFα, IL-8 and IFN-γ for *2*.II.1 (sample obtained at a time of remission, yet no sample was available during disease activity for comparison) and high levels for *2*.III.1 (sample obtained during active keratitis) compared to healthy controls. Not surprisingly, pro-inflammatory cytokines determined on the lacrimal fluid of patient *2*.III.1 were higher compared to a matched healthy control (**Supplementary E4**)

### Immunologic findings of NFKB1 V263L and c.1638-2A>G affected individuals

**Figure 5:**
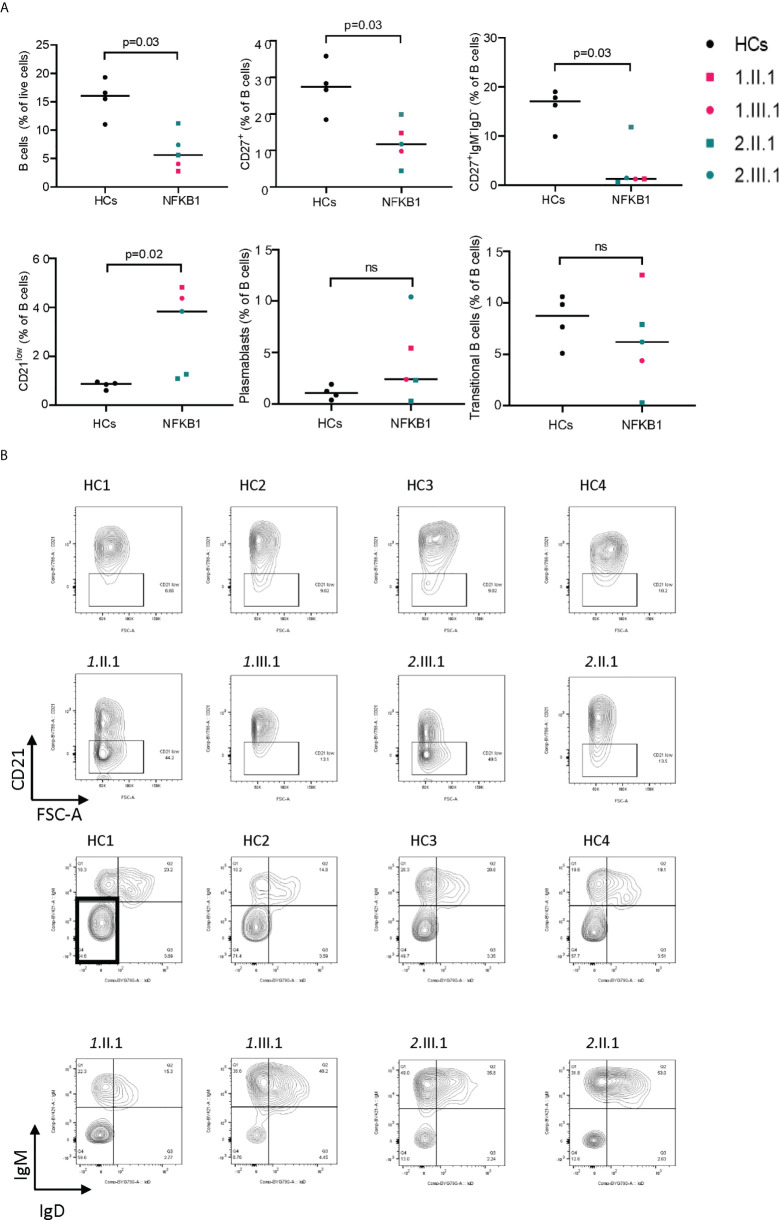
B-cell immunophenotype in NFKB1 affected carriers. **(A)** Relative percentage of cells (gated from indicated population) in NFKB1 affected carriers and healthy controls. **(B)** Gating shown for CD21 low B cells (upper panel) and switched memory B cells (lower panel) for HCs and patients. ns, not significant.

We assessed the B cell compartment of affected patients using flow cytometry (**Supplementary E5**). Results of routine clinical immunophenotyping are found in **Table 2**. In general, affected patients had lower B (CD19+), memory B (CD27+), switched memory B (CD27+IgM-IgD-), and high CD21low B cells (**Figure 5**). In our patients, no differences were seen within the general NK cell compartment (**Supplementary E6**, **E7**), and maturation (assessed by CD27 and CD57) on CD56dimCD16+ cells did not differ from healthy controls (**Supplementary E6**, **E6**). In addition, the monocyte compartment (**Supplementary E6**, **E8**) was similar to healthy controls in subsets (classical-intermediate-nonclassical) and activation markers (CD86, CD38 on classical monocytes).

### NFKB1 V263L exhibits altered protein interactions

**Figure 6:**
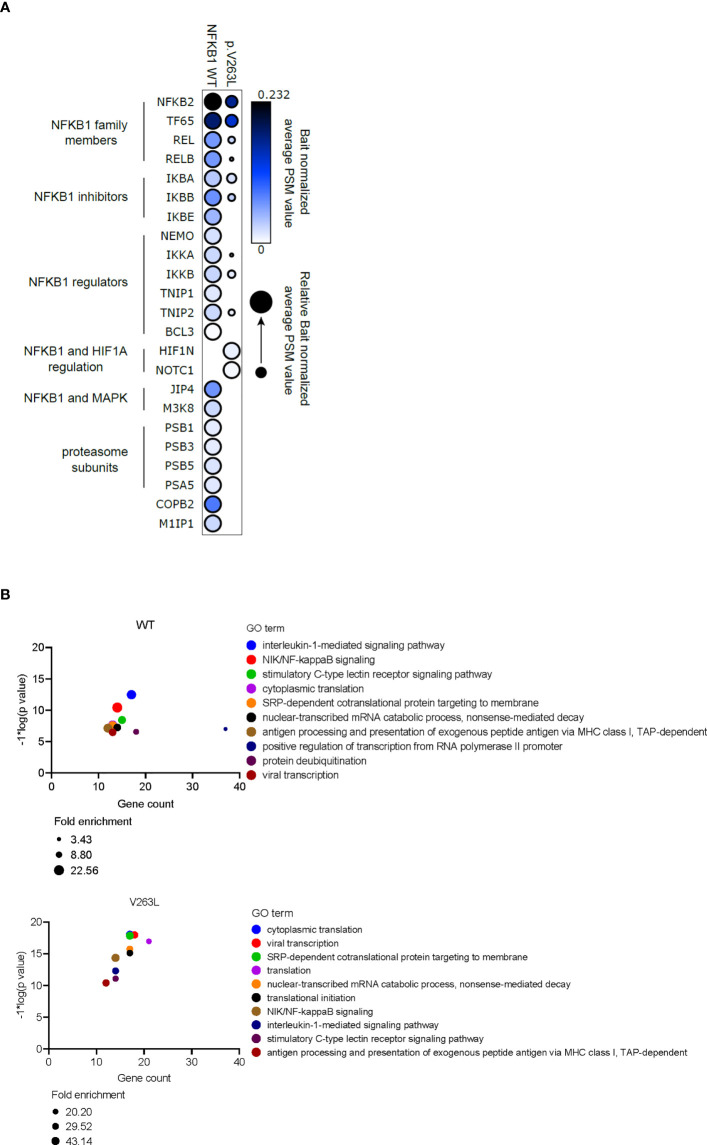
Altered protein interactions of V263L NFKB1. **(A)** Dotplot showing bait normalized PSM values for AP-MS interactors of WT and V263. Circle size depicts the relative normalized PSM values for shared interactors. Triplicate samples were individually bait normalized by the NFKB1 PSM values and the normalized average PSM values were used to make dotplot with ProhitsViz. **(B)** Top 10 most significant GO biological process terms for WT and V263L BioID interactors, using DAVID GO analysis. Gene count, fold enrichment score and -log*1(p value) for every top10 GO term is shown.

To study the changes in protein interactions, caused by p.V263L, we analyzed the stable (AP-MS) and proximal (BioID) interactomes of V263L and WT NFKB1 by generating stable inducible cell lines, expressing the studied NFKB1 constructs tagged with both HA affinity tag and BirA biotin ligase tag. The AP-MS and BioID analysis showed that WT NFKB1 interacted with well-known NFKB1 interactors including the NFKB1 family members, NFKB1 inhibitors and several proteins associated with NFKB1 regulation (**Figure 6**). In both analyses, V263L strikingly led to a reduction or complete loss for almost all interactions compared to the WT (**Figure 6A** and **Supplementary E9**). However, the loss of interaction with the proteasome subunits in AP-MS was not confirmed in the BioID analysis and V263L maintained preserved interactions with NFKB2 and TF65. Gained interactions of V263L with hypoxia-inducible factor 1-alpha inhibitor (HIF1N) and NOTCH1 were observed in both analyses. HIF1N hydroxylates Asn678 within ankyrin repeat domains (ARD) of NFKB1 (12, 13). So far, the role of this hydroxylation has not been elucidated (12). NOTCH1 has been linked to the inhibition of NF-κB activity as its N-terminal portion inhibited p50 DNA binding and interacted specifically with p50 subunit, but not p65 of NF-κB (14). Additional interaction partners exclusive to V263L by the BioID analysis, not retrieved in the AP-MS analysis, can be found in **Supplementary E9**. Next, using the BioID interactors from WT and V263L NFKB1, we performed gene ontology (GO) enrichment analysis to investigate in which biological processes they were involved compared to a population database set (total of 19349 genes). **Figure 6B** shows the top 10 gene sets within the biological processes enriched for WT and V263L with p-values, gene counts and fold enrichment values. As expected for WT, interaction proteins are encoded by genes involved in NF-κB signaling, translation and transcription, viral immunity and posttranslational modification. For V263L, 8 out of the 10 most significantly enriched biological processes overlapped with those observed for WT. Although the mutant NFKB1 had a decreased binding affinity towards various proteins involved in NF-κB signaling, seen in the AP-MS (**Figure 6A**) and BioID **(** **Supplementary E9**) analysis, it still interacted with proteins involved in similar biological processes to WT.

## Discussion

We report two families with novel heterozygous *NFKB1* mutations and diverging phenotypes ranging from CVID with infectious susceptibility, lymphoproliferation and inflammatory features to a subclinical phenotype with only immunological abnormalities. One family member in family 2 even had no phenotype or laboratory abnormalities, demonstrating incomplete penetrance. *In silico* prediction tools suggested that both mutations were damaging. The c.787G>C (p.V263L) mutation is located in the RHD of the protein. RHD is required for dimerization, DNA binding and nuclear translocation. Most of the reported pathogenic mutations are located in this domain and may cause p50 haploinsufficiency or affect both p105 and the active p50 (15). Structural modelling of the mutant V263L NFKB1 suggested an unstable protein caused by a disruption of the C-terminal β-barrel. This was confirmed by demonstrating a significant reduction in p105/p50 expression in both affected members. The c.1638-2A>G mutation is located in the splice site before exon 16, and predicted to alter splicing. This led to decreased NFKB1 transcript and protein levels in all NFKB1 mutation carriers. We could not observe an alternatively in frame spliced NFKB1 mutant by PCR, nor Western blot, suggesting that the alternatively spliced mRNA was rapidly degraded by nonsense mediated decay. Interestingly the phenotypes differed significantly intrafamilial. In the first family, the index patient was most severely affected in contrast to her daughter who met the criteria of CVID but displayed only mild infections. NFKB1 transcript levels were higher in the daughter which might suggest a compensatory mechanism by modifier (epi)genes (enhancing the transcription of WT NFKB1) or allelic exclusion where the transcription of WT *NFKB1* is preferred over mutant. In the second family, the non-affected carrier had the highest transcript and protein levels of NFKB1, again suggesting that modifier (epi)genes could play a role in disease manifestation. Eventually, this could introduce a gene dosage effect, contributing to phenotypical differences despite the presence of an identical mutation. Analyzing IκBα degradation showed normal degradation in both monocytes and lymphocytes using different stimuli for canonical NF-κB pathway activation, demonstrating that our patients did not have any upstream defect.

Because NF-κB is involved in the regulation of the NLRP3 inflammasome (10) and IFN stimulated genes (16) and previous reports have demonstrated excessive inflammasome activation in *NFKB1* patients with inflammatory phenotypes (17), we assessed NLRP3 inflammasome activity and ISG expression in affected patients. In all patients with inflammatory phenotypes we could demonstrate excessive NLRP3 inflammasome activity and an upregulation of the ISG score compared to matched healthy controls. This confirms the previous observation that p50 deficiency in macrophages can promote IL-1β production and lead to tissue inflammation (17). The exact underlying mechanism of NLRP3 activation is still unclear, but NF-κB has a dual (both positive and negative) role in inflammasome regulation. Although one expects NFKB1 haploinsufficiency to result in attenuated immune responses, evidence exists that NFKB1 can also restrain inflammatory responses, hence explaining the inflammatory features in patients with NFKB1 haploinsufficiency (17, 18). A recent study investigated NFKB^-/-^ THP-1 cells as a model of NFKB1^-/-^ human macrophages and demonstrated that activated NFKB1^-/-^ macrophages are more pro-inflammatory than WT controls and express elevated levels of TNFα, IL6, and IL1β, but also have reduced expression of co-stimulatory factors important for the activation of T cells and adaptive immune responses such as CD70, CD83 and CD209 (18). In addition, increased gene expression of components of the interferon response, including Interferon Beta 1, Interferon Lambda 1, and Interferon Alpha And Beta Receptor Subunit 2, in LPS-stimulated NFKB1^-/-^ cells relative to WT controls also identified NFKB1 as a regulator of interferon pathways (18). In concordance, some patients with NFKB1 haploinsufficiency showed increased NLRP3 inflammasome activity in their macrophages upon stimulation with lipopolysaccharide (LPS) and adenosine 5’-triphosphate (17). Finally, *Nfkb1* knockin mice that express p50 but not its precursor, the IκB-like molecule p105, displayed aberrant NF-κB activation and spontaneously developed colitis (19). We confirmed these key observations by demonstrating increased NLRP3 inflammasome activity and upregulation of interferon stimulated genes in our patients who exhibited inflammatory features.

NFKB1 haploinsufficiency is mainly characterized by disturbances in the B cell immunophenotype (6, 20). Most patients (>50%) reported have reduced total and memory B cells and switched memory B cells (6). A recent report describes the presence of CD21low B cell as a potential biomarker to discriminate clinically affected from unaffected members (20) and another study correlated expansion of CD21low B cells with autoimmune cytopenia and lymphoproliferation (6). Affected NFKB1 haploinsufficiency patients in our cohort also had low (memory) B cells, switched memory B cells and relatively high numbers of CD21 low B cells compared to healthy controls.

Impaired NK cell maturation, defective cytotoxicity and reduced IFN-γ secretion has been observed in some NFKB1 patients (21). In our patients, no effect on NK cell maturation (CD57, CD27 expressivity) was noticed. However, we did not provide in depth functional testing of the NK cell compartment, hence the functionality of NK cells could still be impaired.

Finally, we detected altered protein interaction profiles between WT and mutant V263L NFKB1 consistent with a hypo- rather than neomorphic effect of V263L. Loss of interaction between V263L and other NF-κB transcription factors, regulators and inhibitory molecules can impact downstream outcome of NF-κB signaling in both ways. For example, the loss of interaction with RelB which is activated by canonical NF-κB signaling might indicate reduced canonical pathway activation in mutant cells (22). However, in dendritic cells and macrophages, p50:RelB dimers regulate expression and activation of certain anti-inflammatory genes, hence a lost interaction in these cells could prevent the restraining of inflammatory responses (23).

In conclusion, we validated two novel variants in *NFKB1* to be the cause of CVID in two families characterized by variable and incomplete penetrance. Determining the expression of p50/p105 in primary cells (PBMCs) was a first step for assessing the pathogenicity of the variants and they appeared to cause mRNA and/or protein instability leading to haploinsufficiency. Both variants were seen in conjunction with a hyperinflammatory phenotype linked with excessive NLRP3 inflammasome activation and upregulation of ISG. These results highlight the importance of p50/p105 for proper immune function and control of inflammation.

## Data availability statement

The data is accessible in GenBank under the accession numbers: OP183409, OP183410, OP183411, OP183412, OP183413, OP183414, OP183415.

## Ethics statement

The studies involving human participants were reviewed and approved by Ethics committee of the University of Leuven (s58466). The patients/participants provided their written informed consent to participate in this study.

## Author contributions

FS and RS conceptualized the project. FS, MK, SK, XB, MVar, and RS designed the methods. FS, KDK, MK, SK, and FL performed formal experimental analyses. FS, KDK, MK, SK, FL, MVan, and XB performed investigations. SVL, AC, and KW performed genetic analyses. FS and RS wrote the initial draft of the manuscript. RS supervised the project. All authors revised the manuscript. All authors read and approved the final manuscript.

## Funding

FS (11B5520N) and JN (11C3521N) are fellows of the Fonds Wetenschappelijk Onderzoek - Vlaanderen National Fund for Scientific Research (FWO). RS is FWO senior clinical investigator fellows (1805518N, respectively) and received funding from KU Leuven C1 (C12/16/024). This work was supported by the VIB Grand Challenges Program. MVar was supported by grants from the Academy of Finland (nos. 288475 and 294173), the Sigrid Jusélius Foundation, HiLIFE Fellow funding, Magnus Ehrnrooth Foundation, and the Instrumentarium Research Foundation. MS has received financial support from Helsinki University Hospital Research Funds and from the Foundation of Pediatric Research, Finland. IM is FWO senior clinical investigator, and received funding from KU Leuven C1 (C12/16/024).

## Conflict of interest

The authors declare that the research was conducted in the absence of any commercial or financial relationships that could be construed as a potential conflict of interest.

## Publisher’s note

All claims expressed in this article are solely those of the authors and do not necessarily represent those of their affiliated organizations, or those of the publisher, the editors and the reviewers. Any product that may be evaluated in this article, or claim that may be made by its manufacturer, is not guaranteed or endorsed by the publisher.

## Supplementary material

Supplementary Figure 1
**(A)** Sanger sequence traces, **(B)** MAF vs CADD score for all heterozygous NFKB1 mutations reported in gnomAD, studied mutations are indicated by a red dot **(C)** schematic representation of the structural modelling on the p.V263L mutant (red) (described in methods section) and RMSD plot for the wild type (grey) and V263L mutant (red) during a 200 ns MD. RMSD, root-mean-squared deviation; MD, molecular dynamics.

Supplementary Figure 2 Protein expression analysis of p105/p50 in NFKB1 mutation affected and unaffected carriers. Blots from n=6 independent experiments.

Supplementary Figure 3 Alternative mRNA splicing by the c.1638-2A>G mutation. Agarose gel showing the amplified PCR fragment (expected size 750 bp) using primers from exon 14-19 for patient 2.II.1 and 2 healthy controls. No alternative spliced product was detected (representative of n=2 experiments).

Supplementary Figure 4 Cytokine measurement on lacrimal fluid by multiplex analysis (n=1 experiment).

Supplementary Figure 5 Gating strategy for the B cell compartment (live CD14-CD3-CD19+ cells), shown on a representative example (n=1 experiment). Following this gating transitional B cells (CD24+CD38+), CD21low B cells (CD21low), memory B cells (CD27+) and switched memory B cells (CD27+IgM-IGD-) were identified.

Supplementary Figure 6 Gating strategy for the NK (live CD19-CD3-CD14-CD56+) and monocyte (live CD19-CD3-CD56-HLA-DR+) compartment, shown on a representative sample (n=1 experiment).

Supplementary Figure 7 Distribution of the NK cell compartment in HCs and NFKB1 affected individuals (n=1 experiment).

Supplementary Figure 8 Distribution of the monocyte cell compartment in HCs and NFKB1 affected individuals (n=1 experiment).

Supplementary Figure 9 Results of the BioID analysis of WT and V263L NFKB1 with the average bait normalized PSM values given for every protein.

Supplementary Table 1 List of fluorochrome antibodies used for immunophenotyping the main T cell subsets (CD3, CD4, CD8+ T cells), B cells, NK cells and monocytes.
