# Supplementary figures and images for: Common variable immunodeficiency in two kindreds with heterogeneous phenotypes caused by novel heterozygous NFKB1 mutations
Source: Front Immunol. 2022 Sep 20;13:973543. doi: 10.3389/fimmu.2022.973543 (PMC9530060; doi:10.3389/fimmu.2022.973543)

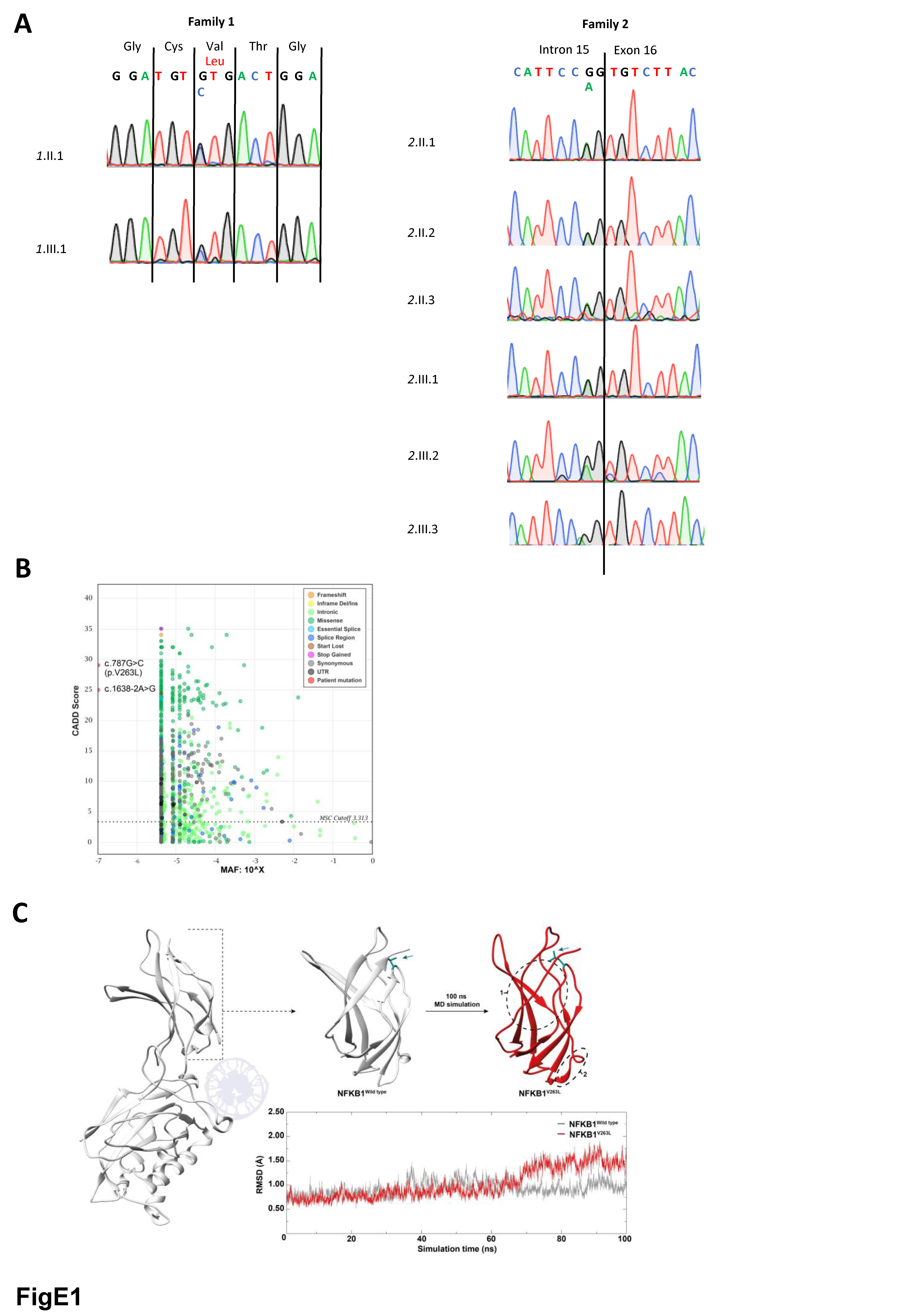

Supplement: Supplementary Figure 1 — (A) Sanger sequence traces, (B) MAF vs CADD score for all heterozygous NFKB1 mutations reported in gnomAD, studied mutations are indicated by a red dot (C) schematic representation of the structural modelling on the p.V263L mutant (red) (described in methods section) and RMSD plot for the wild type (grey) and V263L mutant (red) during a 200 ns MD. RMSD, root-mean-squared deviation; MD, molecular dynamics. [file Image_1.tif]

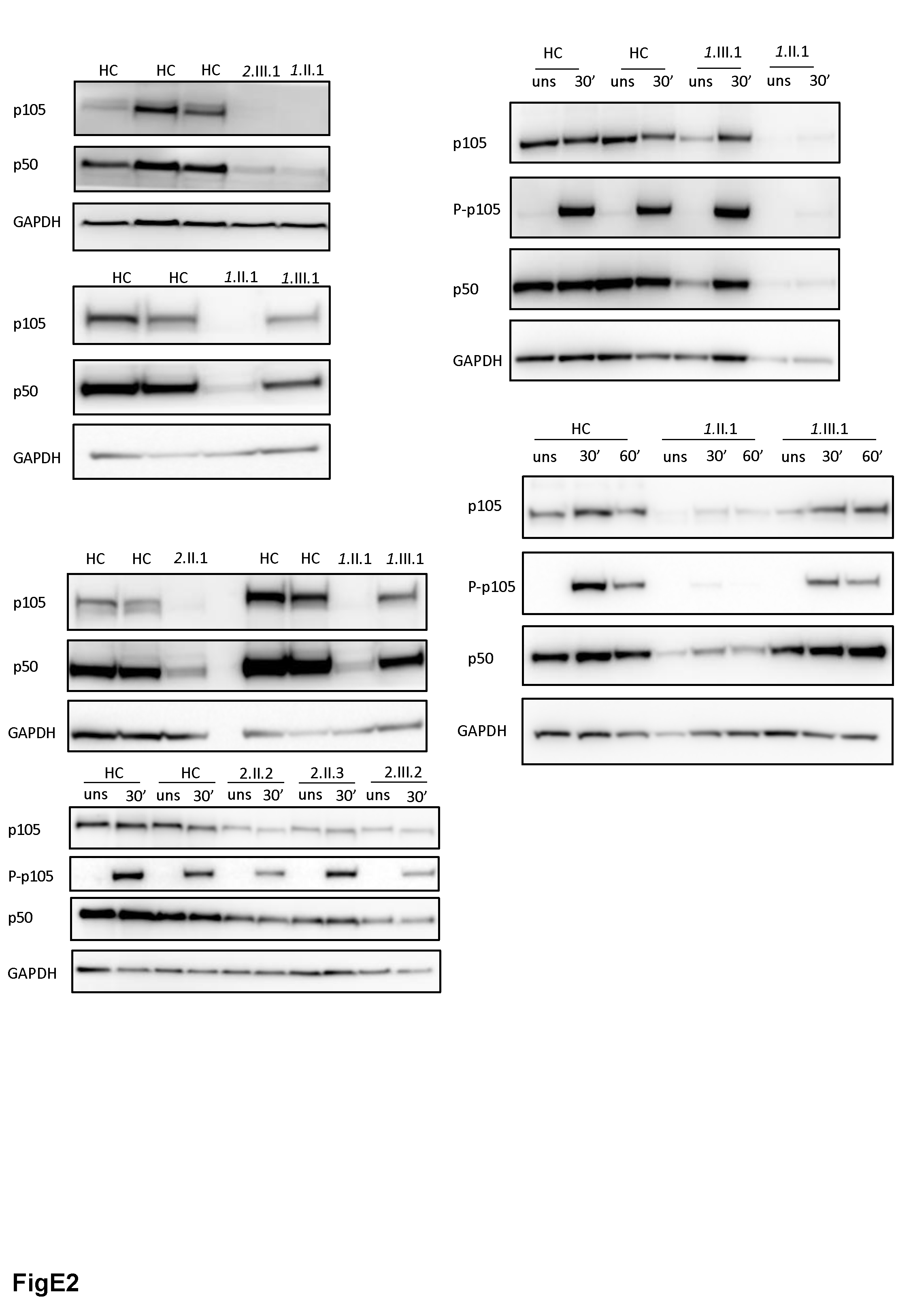

Supplement: Supplementary Figure 2 — Protein expression analysis of p105/p50 in NFKB1 mutation affected and unaffected carriers. Blots from n=6 independent experiments. [file Image_2.tif]

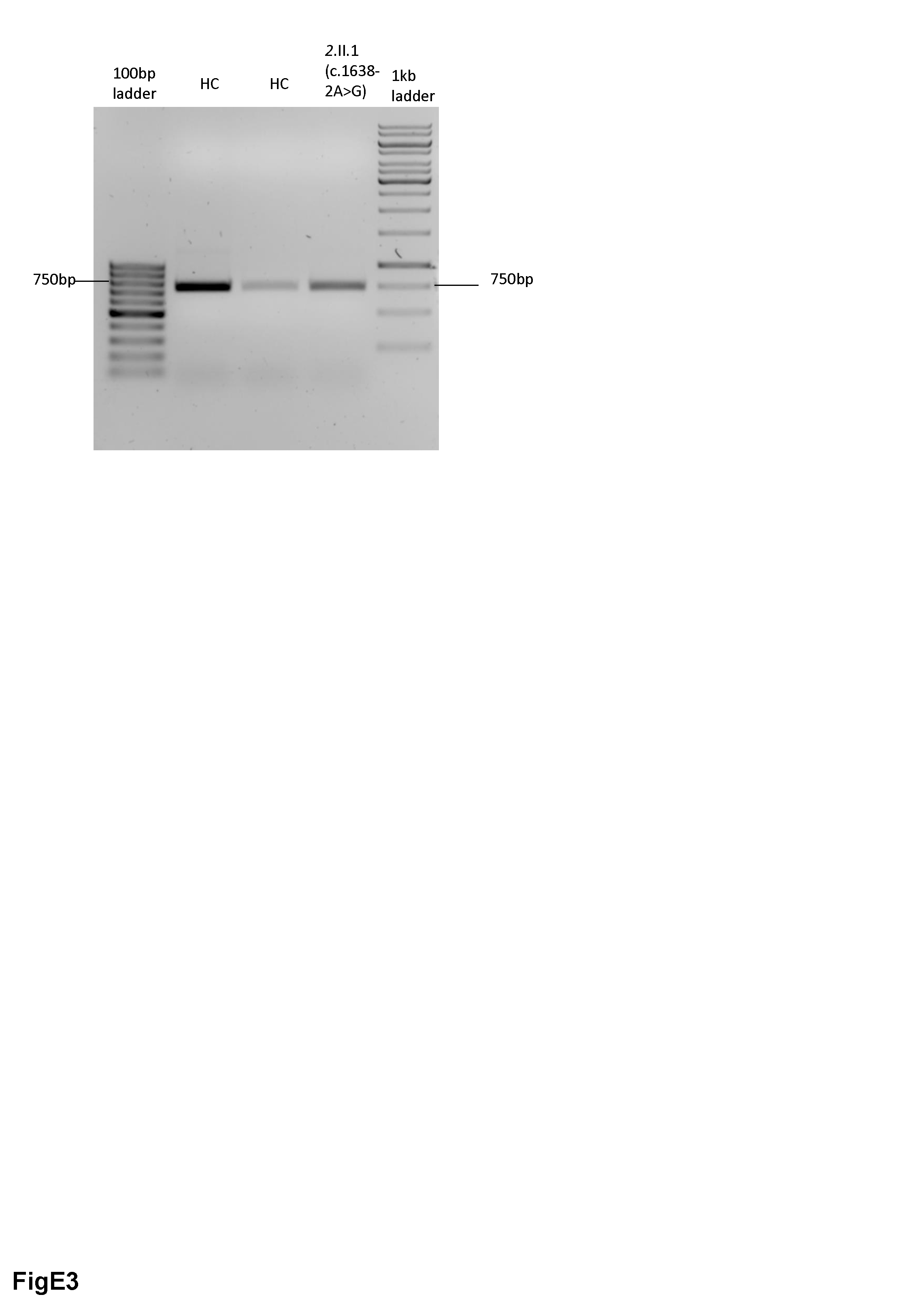

Supplement: Supplementary Figure 3 — Alternative mRNA splicing by the c.1638-2A>G mutation. Agarose gel showing the amplified PCR fragment (expected size 750 bp) using primers from exon 14-19 for patient 2.II.1 and 2 healthy controls. No alternative spliced product was detected (representative of n=2 experiments). [file Image_3.tif]

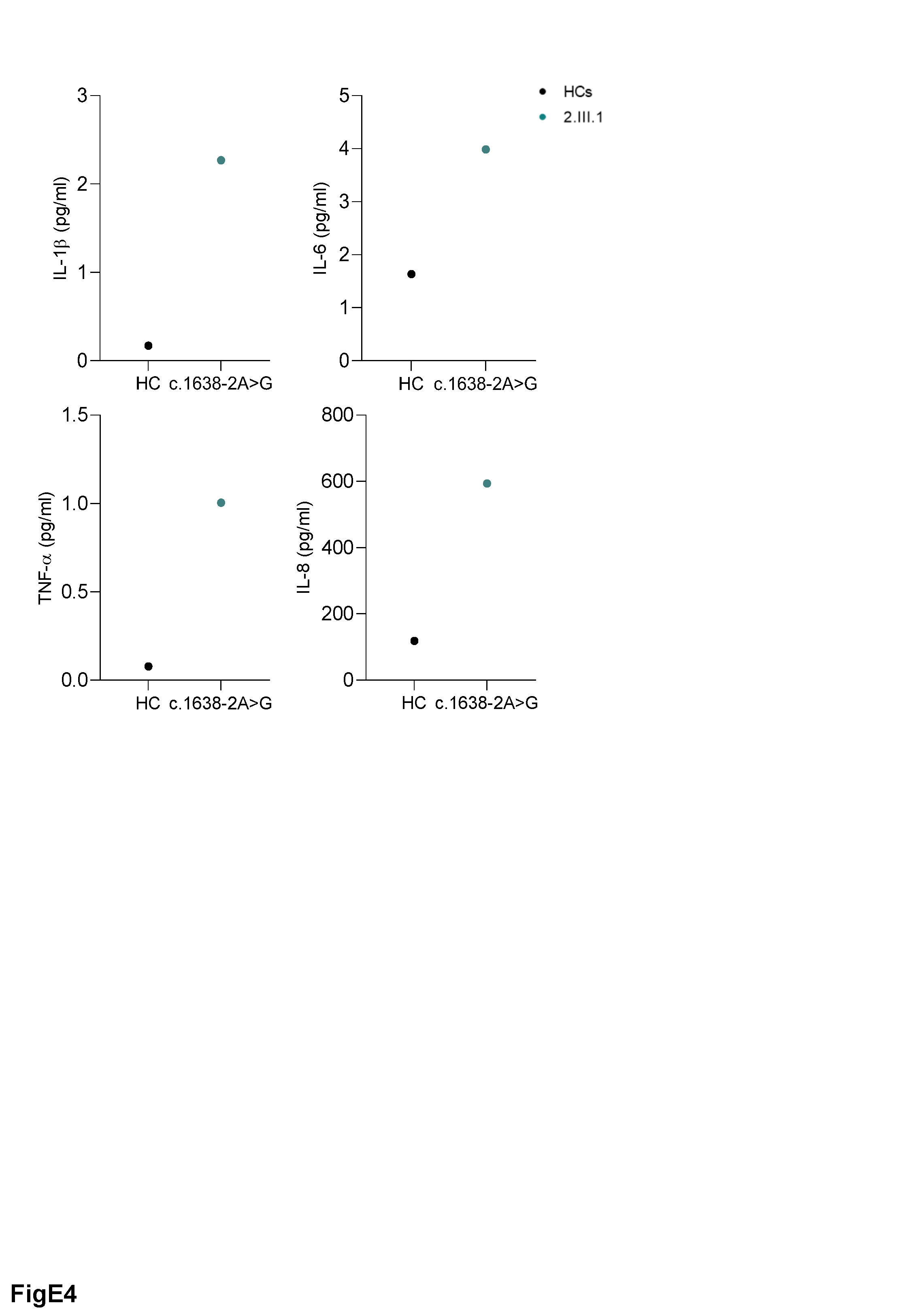

Supplement: Supplementary Figure 4 — Cytokine measurement on lacrimal fluid by multiplex analysis (n=1 experiment). [file Image_4.tif]

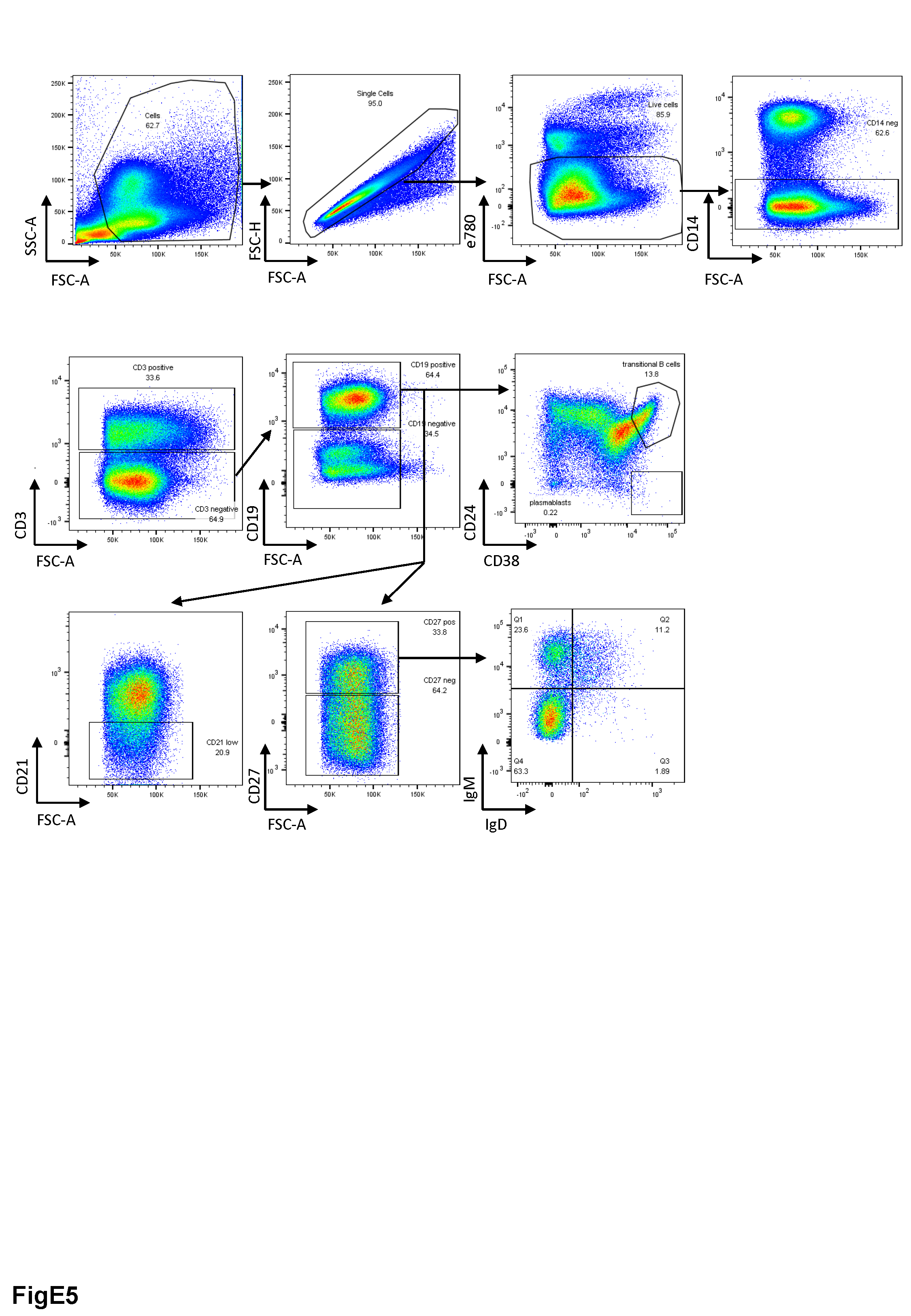

Supplement: Supplementary Figure 5 — Gating strategy for the B cell compartment (live CD14-CD3-CD19+ cells), shown on a representative example (n=1 experiment). Following this gating transitional B cells (CD24+CD38+), CD21low B cells (CD21low), memory B cells (CD27+) and switched memory B cells (CD27+IgM-IGD-) were identified. [file Image_5.tif]

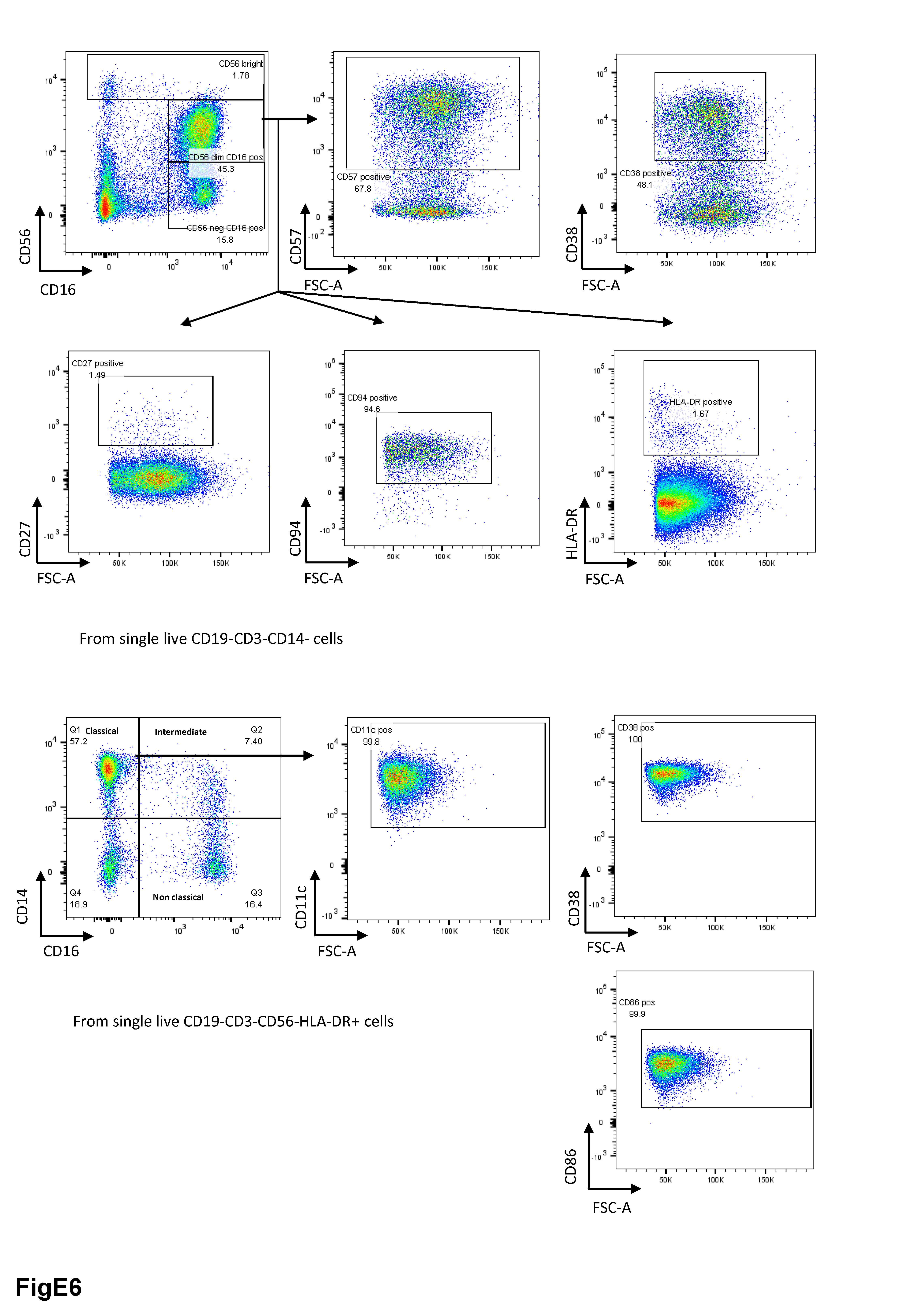

Supplement: Supplementary Figure 6 — Gating strategy for the NK (live CD19-CD3-CD14-CD56+) and monocyte (live CD19-CD3-CD56-HLA-DR+) compartment, shown on a representative sample (n=1 experiment). [file Image_6.tif]

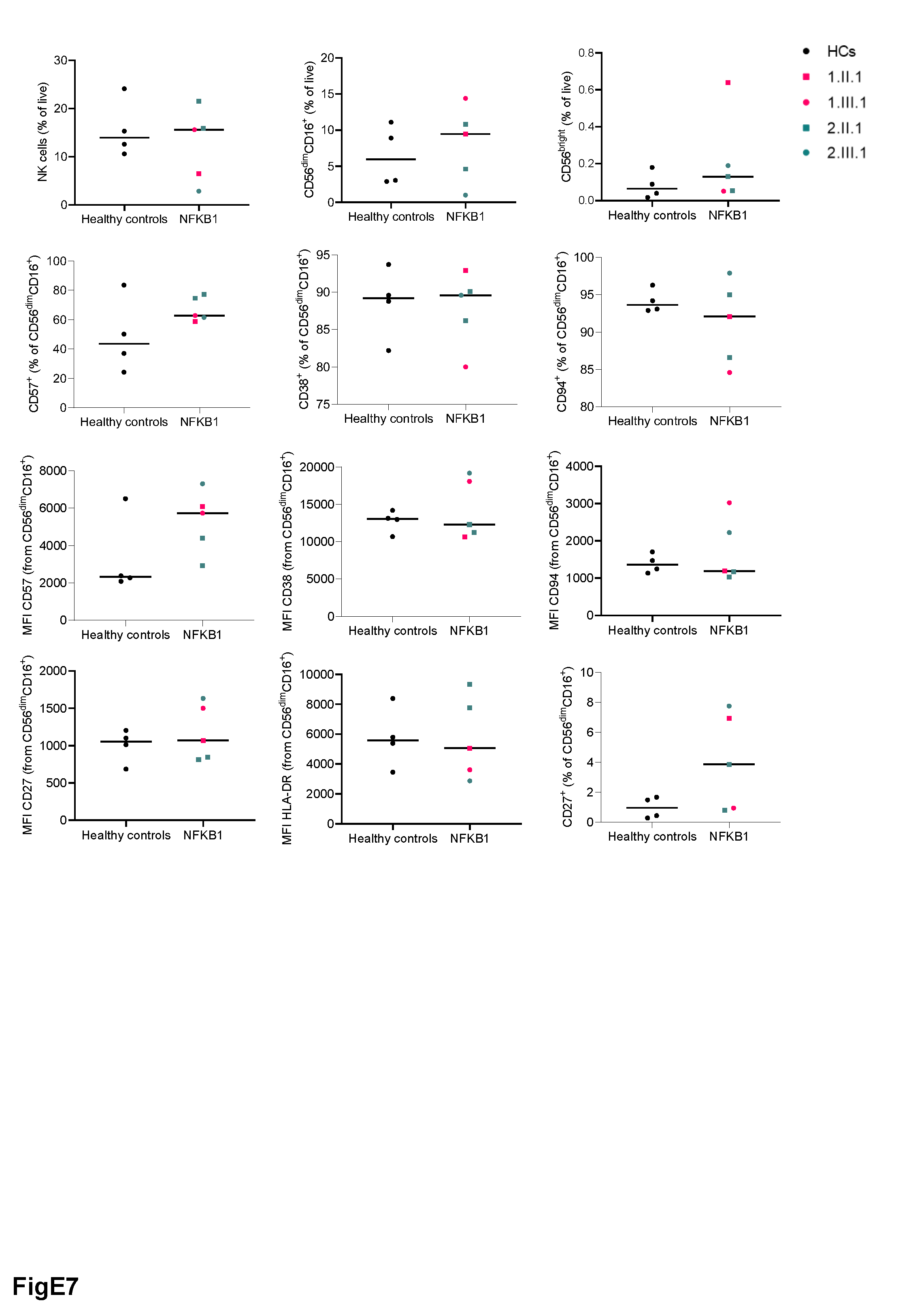

Supplement: Supplementary Figure 7 — Distribution of the NK cell compartment in HCs and NFKB1 affected individuals (n=1 experiment). [file Image_7.tif]

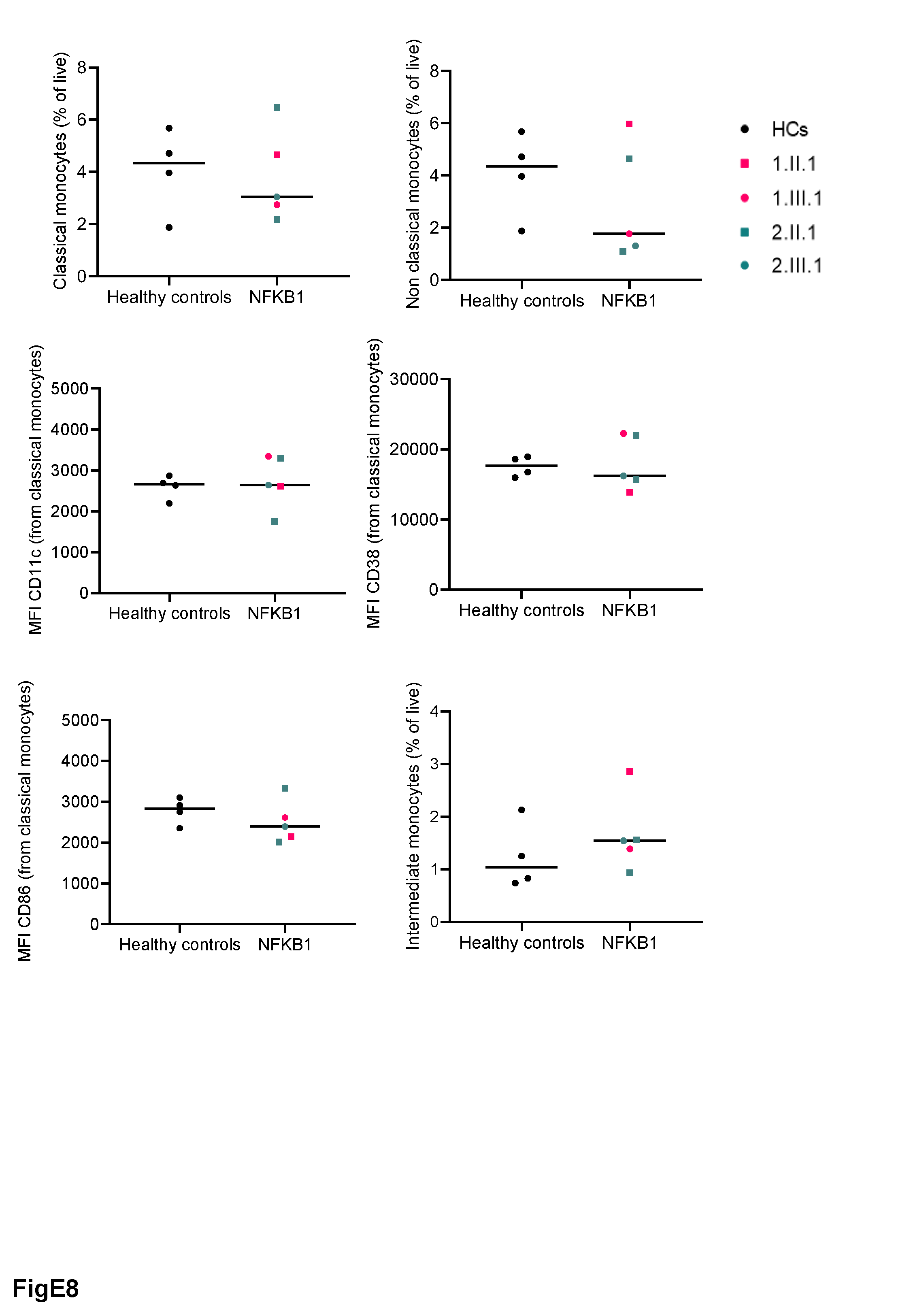

Supplement: Supplementary Figure 8 — Distribution of the monocyte cell compartment in HCs and NFKB1 affected individuals (n=1 experiment). [file Image_8.tif]

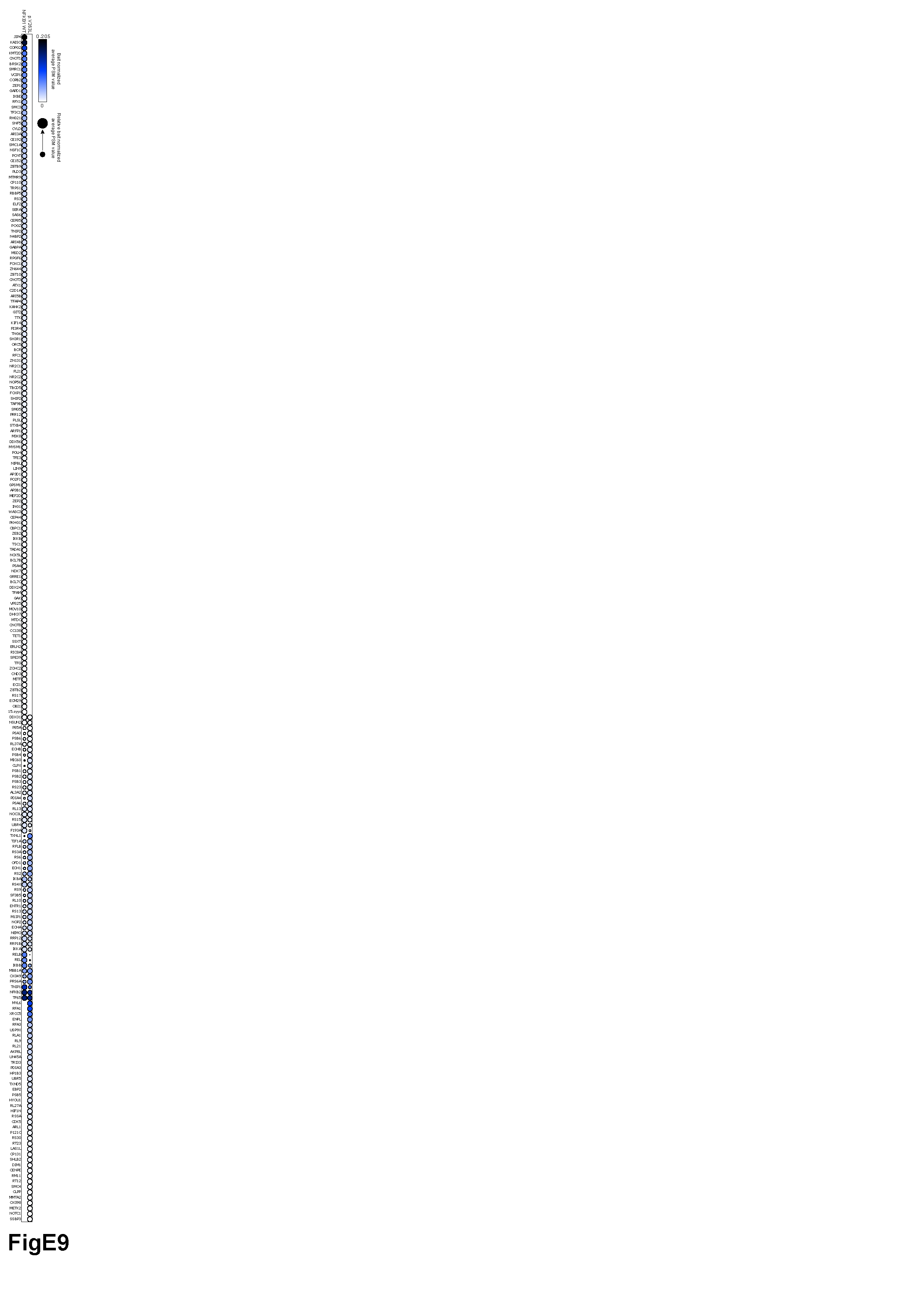

Supplement: Supplementary Figure 9 — Results of the BioID analysis of WT and V263L NFKB1 with the average bait normalized PSM values given for every protein. [file Image_9.tif]
