# Supplementary material for: Common variable immunodeficiency in two kindreds with heterogeneous phenotypes caused by novel heterozygous NFKB1 mutations
Source: Front Immunol. 2022 Sep 20;13:973543. doi: 10.3389/fimmu.2022.973543 (PMC9530060; doi:10.3389/fimmu.2022.973543)
Supplement: Supplementary Table 1 — List of fluorochrome antibodies used for immunophenotyping the main T cell subsets (CD3, CD4, CD8+ T cells), B cells, NK cells and monocytes. [file Table_1.docx]

| **Target** | **Fluorochrome** | **Clone** | **Company** |
| --- | --- | --- | --- |
| anti-CD8 | BUV805 | SK1 | BD bioscience |
| anti-CD4 | BUV496 | SK3 | BD bioscience |
| anti-CD86 | BUV737 | 2331 FUN-1 | BD bioscience |
| anti-CD141 | BUV615-P | 1A4 | BD bioscience |
| anti-CD56 | BUV563 | NCAM 16.2 | BD bioscience |
| anti-CD16 | BUV395 | 3G8 | BD bioscience |
| anti-CD123 | BB660-P | 7G3 | BD bioscience |
| anti- CD80 | BB630 | L307.4 | BD bioscience |
| anti-CD21 | BV785 | B-ly4 | BD bioscience |
| anti-CD27 | BV750-P | L128 | BD bioscience |
| anti-BAFF-R | BV650 | 11C1 | BD bioscience |
| anti-CD94 | BV605 | HP-3D9 | BD bioscience |
| anti-CD40 | APC-R700 | 5C3 | BD bioscience |
| anti-CD3 | PerCP-Vio700 | REA613 | Miltenyi Biotec |
| anti-CD57 | FITC | TB01 | eBioscience |
| anti-CD14 | PE-Cy5.5 | TuK4 | eBioscience |
| anti-CD24 | BV711 | ML5 | BioLegend |
| anti-CD19 | BV510 | HIB19 | BioLegend |
| anti-HLA-DR | BV570 | L243 | BioLegend |
| anti-IgM | BV421 | MHM-88 | BioLegend |
| anti-CD11c | APC | 3.9 | BioLegend |
| anti-CD38 | PE/Dazzle 594 | HB-7 | BioLegend |
| anti-CD10 | PE-Cy5 | HI10a | BioLegend |
| anti-IgD | PE-Cy7 | IA6-2 | BioLegend |

**Table E1** List of fluorochrome antibodies used for immunophenotyping the main T cell subsets (CD3, CD4, CD8+ T cells), B cells, NK cells and monocytes.
